# Visual percepts modify iconic memory in humans

**DOI:** 10.1038/s41598-018-31601-4

**Published:** 2018-09-06

**Authors:** Yoichi Sugita, Souta Hidaka, Wataru Teramoto

**Affiliations:** 1 Department of Psychology, Waseda University, 1-24-1 Toyama, Shinjuku, Tokyo 162-8644 Japan; 2 Department of Psychology, Rikkyo University, 1-2-26, Kitano, Niiza-shi, Saitama 352-8558 Japan; 3 Department of Psychology, Kumamoto University, 2-40-1 Kurokami, Chuo-ku, Kumamoto, 860-8555 Japan

## Abstract

Our visual system briefly retains a trace of a stimulus after it disappears. This phenomenon is known as iconic memory and its contents are thought to be temporally integrated with subsequent visual inputs to produce a single composite representation. However, there is little consensus on the temporal integration between iconic memory and subsequent visual inputs. Here, we show that iconic memory revises its contents depending upon the configuration of the newly produced single representation with particular temporal characteristics. The Poggendorff illusion, in which two collinear line segments are perceived as non-collinear by an intervening rectangle, was observed when the rectangle was presented during a period spanning from 50 ms before to 200 ms after the presentation of the line segments. The illusion was most prominent when the rectangle was presented approximately 100 to 150 ms after the line segments. Furthermore, the illusion was observed at the center of a moving object, but only when the line segments were presented before the rectangle. These results indicate that the contents of iconic memory are susceptible to the modulatory influence of subsequent visual inputs before being translated into conscious perception in a time-locked manner both in retinotopic and non-retinotopic, object-centered frames of reference.

## Introduction

Our visual system sequentially receives an enormous amount of visual inputs. Given the limited capacity of our spatial and temporal processing, the visual system cannot handle these inputs at the moment they appear. Rather, the visual system briefly retains a trace of a stimulus after it disappears. This is called iconic memory, which is thought to be a surrogate for the original stimulus^1,2^. The capacity of iconic memory is shown to be limited^1,3^. Iconic memory decays over time^4^, but the contents of the iconic memory are reported to be temporally integrated with subsequent visual inputs to produce a single composite perceptual representation^5,6^.

The temporal integration processes between iconic memory and subsequent visual inputs have been a matter of debate^7,8^. The process had been investigated mainly by dichotomous probability; researchers examined whether iconic memory and subsequent visual inputs could be integrated within a certain temporal range^5–8^. Recently, visual systems have been reported to flexibly revise moment’s perceptual contents in accordance with the subsequent visual inputs or events^9^, but the temporal binding process for this visual postdictive process remains controversial^10^. Although it has been pointed out that the integration of visual features changes from sensory coding to memory stage^11^, the temporal profile of the integration between iconic memory and subsequent visual inputs for the representation of a single object remains unclear.

**Figure 1:**
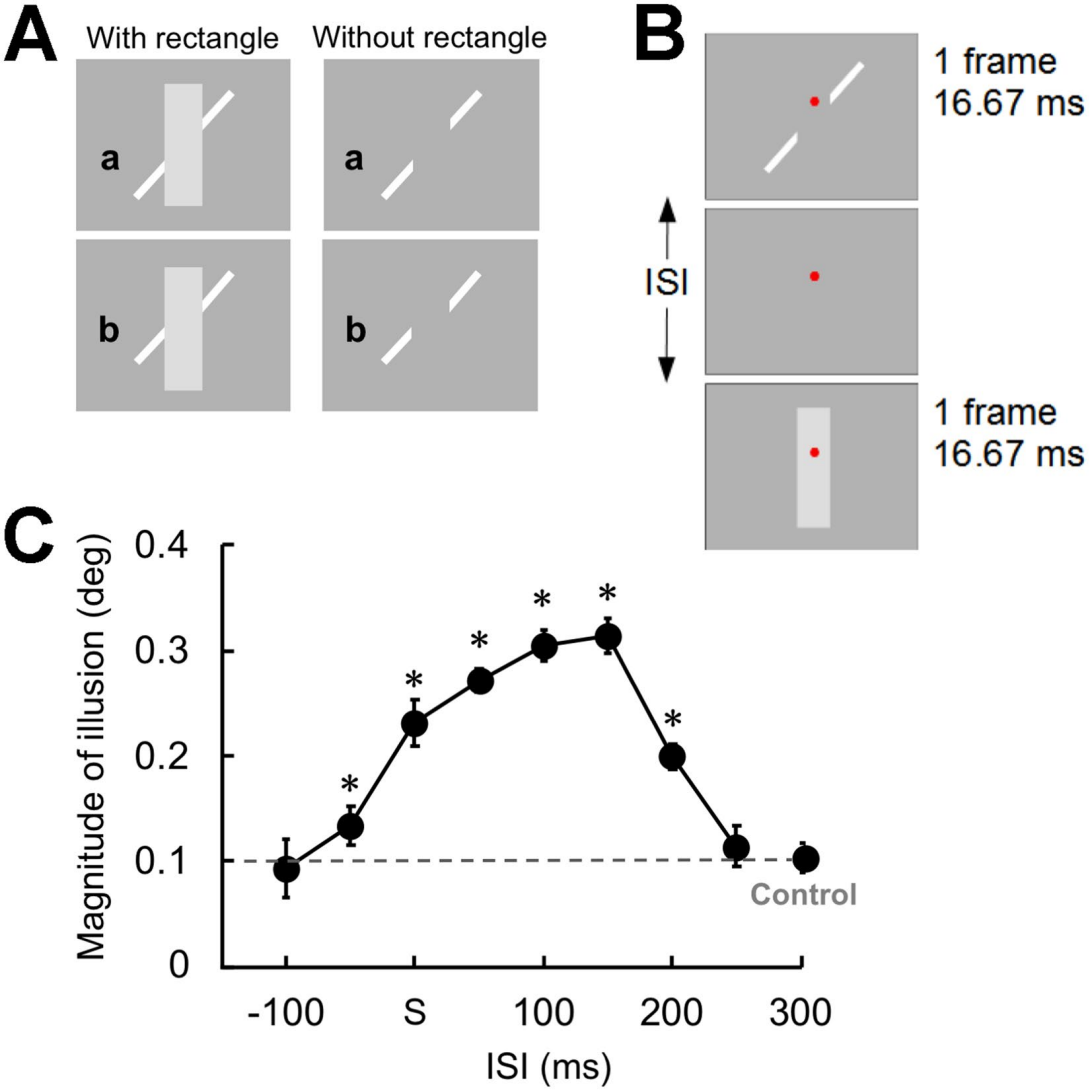
(**A**) Poggendorff figure. In the upper panels, the line segment on the lower left side (a) appears non-collinear with the line segment on the upper right side with a rectangle (left); however, without the rectangle it is obvious that these line segments are actually collinear (right). In the bottom panels, the line segment (b) appears to be collinear with the other line segment with the rectangle (left); however, these line segments are non-collinear (right). (**B**) Sequential presentation of the Poggendorff parts in Experiment 1. Two line segments and a rectangle, all of which are components of the Poggendorff illusion, were presented sequentially. The duration of each component was 16.66 ms. (**C**) The magnitude of the illusion is plotted against the ISI between the line segments and the rectangle. The dashed line represents the point of subjective continuity obtained from the control condition. The letter ‘s’ denotes that the line segments and the rectangle were presented simultaneously. Asterisks denote significant differences compared with the control condition (*p* < 0.05 with Holm’s correction). Error bars denote the SEM (N = 7).

Here, we explore the manner in which iconic memory is integrated with subsequent visual inputs to represent a single object representation across time. To this end, we utilized the Poggendorff figure as the visual stimulus. In this figure, an oblique line is interrupted by the edge of an intervening rectangle, and the two separated but collinear oblique line segments appear non-collinear (Fig. 1A). We presented two oblique line segments and the intervening rectangle briefly and sequentially with various inter-stimulus intervals (ISI) and measured the magnitude of the illusion. The results suggest that the iconic memory revises its contents depending upon the configuration of the newly produced single representation with particular temporal characteristics.

## Results

### Experiment 1: Temporal profile of the magnitude of integration between iconic memory and subsequent visual inputs

We first investigated the temporal profile of the integration between iconic memory and subsequent visual inputs. Two oblique line segments and a rectangle were very briefly and sequentially presented with various ISIs. If the iconic memory was a surrogate of the original stimulus and integrated with subsequent visual inputs to form a single representation, the separated line segments should appear non-collinear even when the segments and the intervening structure were presented sequentially with an appropriate ISI.

To minimize the occurrence of a possible afterimage, the visual stimuli were presented very briefly on a cathode ray rube (CRT) display in a quiet dark room. Observers were asked to continually look at a red fixation point. Each trial began after the observer pressed a button. One second later, the upper right and the lower left oblique line segments were presented for 16.67 ms (only one frame). The position of the lower left segment varied from 0.087° below to 0.435° above the objective continuation of the upper right segment in steps of 0.087°. The intervening rectangle was also presented for 16.67 ms. The ISI between presentation of the line segments and the rectangle changed from −100 ms (whereby the rectangle was presented first) to 250 ms (Fig. 1B). A control trial in which only line segments were presented was also included. The position of the lower left segment and the ISI were selected randomly from trial to trial. The observers were asked to judge whether the lower left segment was above or below the objective continuation of the upper right segment. The magnitude of misalignment indicates the strength of the illusion. To determine the amount of illusory misalignment that corresponds to a point of subjective continuation, we estimated the 50% point (the point of subjective continuity) by fitting a cumulative normal distribution function to each individual’s data using a maximum likelihood curve fitting technique (Supplementary Figure S1).

The illusory misalignment was observed when the intervening rectangle was presented from 50 ms before to 200 ms after the presentation of the line segments (Fig. 1C). It was evident that the temporally separated rectangle induced the illusory misalignment, since the illusion was not observed in the control condition. A planned *t*-test with Holm’s correction (*p* < 0.05) between each ISI condition and the control condition revealed that the magnitudes of the illusion with −50 to 200 ms of ISIs were significantly greater than that of the control condition (*ts*(6) > 3.87, *ps* <0.008, *d*s >1.69) but not for ISIs of −100 and 250 ms (*ts*(6) < 0.95, *ps* >0.38, *d*s <0.48). It has been reported that with ISIs shorter than 50 ms, two visual stimuli are fused and integrated into a single unified percept^6,12^, implying that successful integration relies on the visible persistence of the stimuli presented. The illusory misalignment observed in the present study when the rectangle was presented from 50 ms before to 50 ms after the presentation of the line segments would rely on the same visual persistence. However, it is notable that the illusory misalignment was maximal when the rectangle was presented 100 to 150 ms after the line segments. These ISIs were too long for integration based on visible persistence^6,12^. The results of Experiment 1 thus suggest that nearly collinear lines in iconic memory are first represented by two line segments, but that subsequent visual inputs alter the content of the iconic memory to then represent misaligned lines (Supplementary Video S2).

### Experiment 2: The integration of iconic memory and subsequent visual stimuli can occur in a moving object

It was once claimed that the contents of iconic memory are encoded in retinotopic coordinates and that the iconic memory thus cannot hold any useful information under normal viewing conditions when objects or the observer are in motion^8^. However, it has been assumed that cortical networks integrate sensory inputs to create object-centered representations in which the position of the parts of an object are encoded with respect to an origin and axes centered on the object^13,14^. Therefore, visual inputs can be integrated in a non-retinopic, spatiotopic manner against saccadic eye movements and motion^15–18^. Specifically, it has been shown that when an object moves, the motion of its constituent parts is perceived relative to the object rather than its retinotopic coordinates; therefore, the moving object serves as a non-retinotopic, object-centered reference frame for integrating its parts^19–22^. We examined whether the illusory misalignment was observed when the line segments were perceived as parts of a moving object despite the line segments and the intervening rectangle being presented at different retinal positions.

**Figure 2:**
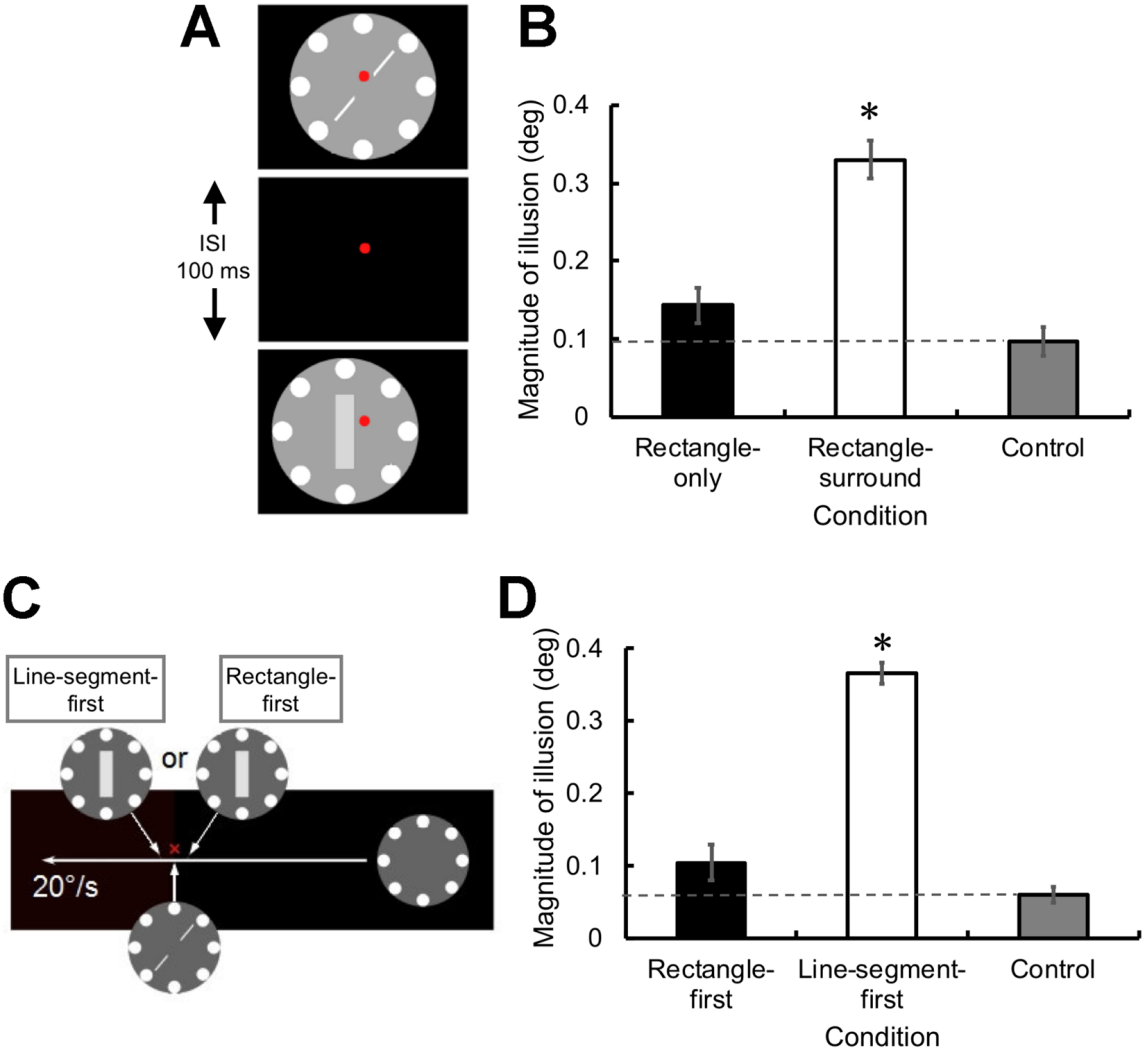
(**A**) Poggendorff parts on an apparent motion stimulus in Experiment 2. Two line segments and a rectangle were presented against a surrounding stimulus with an ISI of 100 ms. The rectangle and the surround stimuli were presented 1° to the left of the original location where the line segments did not abut obliquely on the rectangle. (**B**) The magnitude of illusion in the conditions in which the line segments and the rectangle moved with the surrounding stimulus (rectangle-surround), only the rectangle moved so that the line segment and the surrounding stimulus was stable (rectangle-only), and only the line segments moved with the surrounding stimulus (control). The dashed line represents the point of subjective continuity obtained in the control condition. (**C**) Poggendorff parts on a moving stimulus in Experiment 3. The surrounding stimulus moved from right to left at 20°/sec. Two line segments and a rectangle were presented against a surrounding stimulus with an ISI of 50 ms. (**D**) The magnitude of the illusion in the conditions where the rectangle was presented first, the line segments were presented first, and only the line segments were presented (control). The dashed line represents the point of subjective continuity obtained in the control condition. Asterisks denote a significant difference compared with the control condition (*p* < 0.05 with Holm’s correction). Error bars denote the SEM (N = 7 and 8 for Experiments 2 and 3, respectively).

We presented a surrounding stimulus that served as a moving object. Eight white circles (1.0° in diameter) were drawn equidistant from each other on a gray circle (7.0° in diameter). The line segments and the rectangle were presented against the backdrop of the surrounding stimulus (Fig. 2A). The ISI between the stimuli was 100 ms, and all elements of the stimulus were presented so as to move together^19–21^. One second after the observer pressed the button, the upper right and lower left oblique line segments were presented together with the surrounding stimulus for 16.67 ms. After an ISI of 100 ms, the rectangle with the surrounding stimulus were presented for 16.67 ms at 1° to the left of the original location (rectangle-surround condition). The surrounding stimulus appeared to move from right to left, and the rectangle was not presented at the location where the two line segments abutted obliquely on the edge of the rectangle. However, an illusory misalignment of the line segments may have been observed if the two line segments and the rectangle appeared as parts of the surrounding stimulus and the information of these two stimuli was attributed to the same object^19–22^. Indeed, we observed this to be the case.

When the rectangle and the surrounding stimulus were presented 1° to the left of the original location, the two line segments did not appear to abut obliquely on the edge of the rectangle, indicating that the iconic memory was not completely integrated with the subsequent visual inputs to solely produce a single composite representation. Nevertheless, the line segments appeared to be misaligned (Supplementary Video, S3). A planned *t*-test with Holm’s correction (*p* < 0.05) revealed that the magnitude of the illusion in the rectangle-surround condition was significantly greater than that of the control condition in which only the line segments and the surrounding stimulus were presented as moving (*t*(6) = 7.84, *p* < 0.001, *d* = 2.96) (Fig. 2B). However, when the surrounding stimulus was presented at the same location and only the rectangle was shifted (rectangle-only condition), remarkably, the illusory misalignment decreased significantly to the level observed in the control condition (*t*(6) = 1.64, *p* = 0.15, *d* = 0.62).

### Experiment 3: The integration of iconic memory and subsequent visual stimuli can occur only when the line segments appear after the intervening rectangle in a moving object

The results of Experiment 2 suggest that the content of iconic memory is affected by subsequent visual inputs even when they are not fully integrated with the subsequent visual inputs as a single composite representation if they can be perceived as part of a single moving object. Thus, it is very likely that when information is available to draw an inference about the continuity of a single object along its past trajectory, the present percept may be able to exert a modulatory influence on the iconic memory created on the trajectory in an object-based manner^14^. The third experiment tested this possibility. We presented the surrounding stimulus as moving from right to left at 20°/s. The line segments were presented at the center of the surrounding stimulus for 16.67 ms when the surrounding stimulus passed the center of the display. The rectangle was presented for 16.67 ms when the surrounding stimulus passed 1° to the right or left from the center of the display (Fig. 2C). The ISI between the line segments and the rectangle was 50 ms in both conditions. If iconic memory were susceptible to the modulatory influence of subsequent visual inputs, an illusory misalignment would be observed when the line segments were presented before the rectangle but not in the reverse order. A control condition in which the rectangle was not presented was also included. The illusory misalignment was clearly observed when the line segments were presented before the rectangle (line-segment-first condition), whereas the illusion was not observed in the reverse order (rectangle-first condition) (Supplementary Video S4). A planned *t*-test with Holm’s correction (*p* < 0.05) revealed that the magnitude of the illusion in the line-segment-first condition was significantly greater than that of the control condition (*t*(7) = 17.38, *p* < 0.001, *d* = 6.15). In contrast, the magnitude of the illusion in the rectangle-first condition was comparable with the control condition (*t*(7) = 1.94, *p* = 0.09, *d* = 0.72) (Fig. 2D). These results are extremely close to what would be predicted from the above hypothesis.

## Discussion

The current study investigated the temporal aspects of the integration between iconic memory and subsequent visual inputs by utilizing the magnitude of the Poggendorff illusion as an index of the strength of the integration. When two line segments and an intervening rectangle were presented at the same retinal position where the line segments abutted obliquely on the rectangle’s edge, the illusory misalignment was observed even when the line segments were presented after the rectangle (Experiment 1). A notable point is that the magnitude of the illusion increased linearly with an increase in ISI and peaked at an ISI of 100–150 ms. The illusion occurred reliably even at an ISI of 200 ms. The temporal integration of visual inputs within 50 ms of ISI is likely based on visible persistence^6,12^. The perceptual performance of the temporal integration of visual stimuli is reported to drastically change after an ISI of 100 ms^4,23^, indicating an alteration from percept-percept to image-percept integration^24^. Our findings strongly suggest that the contents of the iconic memory are malleable to subsequent visual inputs especially around 100–150 ms of ISI.

One may consider that the larger magnitude of the illusion at the ISI of 100–150 ms is due to a perceptual masking effect such as backward masking^2,25^. In fact, it has been demonstrated that the masking effect becomes the strongest with a certain temporal delay^26,27^ due to the decay of the target’s perceptual intensity. If backward masking mainly contributed to our results, the participants’ responses would occur in a random, inconsistent manner because the appearance of the target would become uncertain. However, our data clearly demonstrate that the Poggendorff illusion consistently occurred and that the magnitude of the illusion gradually increased with the increment of ISIs. These aspects clearly refute the hypothesis that masking mechanisms can fully explain our data.

The illusory misalignment occurred between the line segments and the intervening rectangle even when they were both spatially (1° in the horizontal direction) and temporally (ISI of 100 ms) separated (Experiment 2), when these stimuli were presented together with the surrounding moving object. Notably, when the rectangle and the surrounding object were presented 1° to the left of the original location, the two line segments did not appear to abut obliquely on the edge of the rectangle, so they did not seem to be a single composite object. Nevertheless, the line segments were clearly perceived as being misaligned (Supplementary Video S3). These observations are highly inconsistent with the view that iconic memory is based on retinotopic coordinates so that it cannot hold any useful information when objects are in motion^8^. However, the illusion was not observed when the spatiotemporal continuity was not maintained between the line segments and the rectangle by the surrounding moving object. Thus, the integration between the contents of iconic memory and subsequent visual inputs and the resulting Poggendorff illusion occur in a non-retinotopic, object-centered manner^18–21^.

When the line segments and the rectangle were presented at the same retinal position and the line segments abutted obliquely on the edge of the rectangle, the illusory misalignment was observed even when the line segments were presented after the rectangle (Experiment 1). However, the misalignment was not observed when the rectangle was presented before the line segments in the situation where the surrounding stimulus was shifted 1° to the left so that the line segments did not abut obliquely on the edge of the rectangle (Experiment 3). Perceptual performance and its possible underlying mechanisms are reported to differ between situations in which temporally separated visual stimuli are presented at the same retinal position and those in which they appear at different spatial positions^18,28,29^. Our visual systems are reported to represent our percept in a postdictive manner in which the subsequent visual inputs affect the percept of the previous inputs. It remains controversial what kind of processing stages, such as perception or memory, contribute to the postdictive nature of visual perception^10^. The current findings clearly suggest that at least two mechanisms may independently contribute to the integrate of temporally discrete visual inputs. One relies on visual persistence and works for ISIs shorter than 50 ms, especially when the visual inputs are at the same retinal position. The other involves the integration of iconic memory previously formed and subsequent visual inputs, which occurs both at a retinotopic location with longer ISIs (peaked at 100–150 ms) and in a non-retinotopic, object-centered frame of reference.

The Poggendorff illusion is assumed to occur due to the mislocalization of the tangent point between lines and a rectangle and the perceptual distortion of the orientation of the lines^30^. Our demonstration (Supplementary Video S3) shows that the Poggendorff illusion occurred even when the Poggendorff figure is not completely represented as a single composite object. This also supports our hypothesis that the perceptual processing related to the Poggendorff illusion could occur due to the integration between the previously formed iconic memory and the subsequent visual inputs. Our current findings suggest that the contents of the iconic memory could be susceptible to the modulatory influence of subsequent visual inputs before they are translated into conscious perception.

Temporal integration processes between iconic memory and subsequent visual inputs have been investigated, mainly focusing on whether iconic memory and subsequent visual inputs could be integrated within a certain temporal range^5–8^ and on how iconic memory persists or decays over time^31–34^. Some studies also reported perceptual illusion^35,36^ and integration^37–39^ based on the temporal integration between iconic memory and subsequent visual inputs. However, temporal profiles or temporal changes in integration processes have not yet been reported. Our study, using the magnitude of the Poggendorff illusion as an index of the strength of integration, newly shows the temporal profiles of the integration between iconic memory and subsequent visual inputs (Experiment 1). Non-retinotopic, object-centered manners of the integration have been reported for a variety of visual features^18^, such as form^20,40^. The current study adds a further understanding that the temporal order of inputs also plays an important role for the integration of the iconic memory and subsequent visual inputs at a non-retinotopic, object-centered frame of reference in order to establish perceptual interpretations (the Poggendorff illusion) of the inputs (Experiment 3). Both the results of Experiments 1 and 3 suggest that perceptual modulations would likely occur specifically when to-be-modulated stimuli are represented in an iconic memory stage. The current findings demonstrate that our visual system revises the contents of iconic memory depending upon the configuration of the newly produced single representation in a time-locked manner.

## Methods

### Ethics statement

The experiments were approved by the local ethics committee of Waseda University and performed in accordance with the Declaration of Helsinki. Informed consent was obtained from each participant before conducting the experiments.

### Participants, apparatus, and stimuli

A total of 22 observers (aged 27–63 years) took part in the experiments (7, 7, and 8 participated in Experiments 1, 2, and 3, respectively). All participants had normal or corrected-to-normal vision and were naive to the experimental conditions. To minimize the occurrence of a possible afterimage, the visual stimuli were presented very briefly on a display in a quiet dark room. Two-dimensional visual stimuli were presented on a 24-inch CRT display (800 × 600 pixel resolution, refresh rate of 60 Hz) at a viewing distance of 1 m. The visual stimuli were components of a Poggendorff figure and consisted of two white line segments (85.0 cd/m^2^) and a light gray rectangle (1° × 3.55°, 38.2 cd/m^2^) (Fig. 1B). The line segments were tilted clockwise at a 40.0° angle. These stimuli were presented on a uniform gray background (22.5 cd/m^2^). The observers were asked to fixate on a red fixation point on the display in a dark room. In Experiments 2 and 3, a surrounding stimulus, which consisted of eight white circles (1.0° in diameter) and a large gray circle (7.0° in diameter), was used to examine the illusion observed when the parts of a Poggendorff figure were presented against a moving object. Eight white circles (85.0 cd/m^2^) were drawn equidistant to each other within a gray circle (22.5 cd/m^2^). The line segments and the rectangle were presented at the center of the surrounding stimulus on a black background (0.01 cd/m^2^).

### Experimental procedure

In Experiment 1, a trial began when the observer pressed a button. After a blank period of 1 s, the upper right and lower left oblique line segments were presented for 16.67 ms (only one frame). The position of the lower left segment varied from 0.087° below to 0.435° above the objective continuation of the upper right segment in steps of 0.087°. The intervening rectangle was also presented for 16.67 ms. The ISI between presentation of the line segments and the rectangle was changed from −100 ms (whereby the rectangle was presented first) to 250 ms (Fig. 1B). A control trial, in which only line segments were presented, was also included. Based on our preliminary observations, we modified the range of the position of the lower left segment in each condition as follows: −0.087° to 0.261° for the ISI of −100 ms, −50 ms, and 250 ms in addition to the control condition; 0.087° to 0.434° for the ISI of 0–150 ms; and 0° to 0.348° for the ISI of 200 ms. The position of the lower left segment and the conditions were selected randomly from trial to trial. The observers were asked to judge whether the lower left segment was above or below the objective continuation of the upper right segment. Five responses were obtained for each condition.

In Experiment 2, we assessed the magnitude of the illusion when the surrounding stimulus served as an apparent motion stimulus. One second after the observer pressed the button, the right and lower left oblique line segments were presented together with the surrounding stimulus for 16.67 ms. After an ISI of 100 ms, the rectangle and the surrounding stimulus were presented for 16.67 ms 1° to the left of the original location. The surrounding stimulus appeared to move from right to left. In this condition, the rectangle was not presented at the location where the two line segments abutted obliquely on the edge of the rectangle (rectangle-surround condition). To examine the effect of the surround motion, another condition where the surrounding stimulus was presented at the same location and only the rectangle was presented 1° to the left of the original location was presented (rectangle-only condition). A control condition in which the rectangle was not presented was also included. Based on our preliminary observations, the ranges of the position of the lower left segment spanned from −0.087° to 0.435° for the rectangle-only and control conditions and from −0.087° to 0.522° for the rectangle-surround condition. Apart from these conditions, the procedures were identical to that of Experiment 1.

In Experiment 3, we examined the magnitude of the illusion when the surrounding stimulus was moving from right to left at 20°/s. The line segments were presented at the center of the surrounding stimulus for 16.67 ms when the surrounding stimulus passed the center of the display. The rectangle was presented for 16.67 ms when the surrounding stimulus passed 1° to the right (rectangle-first condition) or left (line-segment-first condition) of the center of the display. The ISI between the line segments and the rectangle was 50 ms in both conditions. A control condition in which the rectangle was not presented was also included. Based on our preliminary observations, the range of the position of the lower left segment that we used was from −0.087° to 0.348° for the rectangle-first and control conditions and 0.261° to 0.696° for the line segment-first condition. Apart from these conditions, the procedures were identical to that of Experiment 1.

The magnitude of misalignment indicated the strength of the illusion. To obtain a point of subjective continuity, we estimated the 50% point (the point of subjective continuity) by fitting a cumulative Gaussian function to each individual’s data (Supplementary Figure S1) using MATLAB compatible psignifit functions which implements the maximum-likelihood method^41^. We confirmed that the range (mean, SD) of R^2^ in each experiment was 1.00–0.46 (0.91, 0.11), 0.99–0.75 (0.90, 0.07), and 0.99–0.77 (0.91, 0.08) for Experiments 1, 2, and 3, respectively. We performed a planned *t*-test with Holm’s correction (*p* < 0.05) for each experiment.

## Electronic supplementary material

Supplementary video S2

Supplementary video S3

Supplementary video S4

Supplementary Information

## Data Availability

The datasets generated during and/or analyzed during the current study are available from the corresponding author on reasonable request.
