# Supplementary material for: Visual percepts modify iconic memory in humans
Source: Sci Rep. 2018 Sep 6;8:13396. doi: 10.1038/s41598-018-31601-4 (PMC6127220; doi:10.1038/s41598-018-31601-4)
Supplement: Supplementary file 4 — Supplementary Information [file 41598_2018_31601_MOESM4_ESM.docx]

## Supplementary Information

## Visual percepts modify iconic memory in humans

Yoichi Sugita^1^*, Souta Hidaka^2^*, Wataru Teramoto^3^

1. Department of Psychology, Waseda University, 1-24-1 Toyama, Shinjuku, Tokyo, 162-8644 Japan
2. Department of Psychology, Rikkyo University, 1-2-26, Kitano, Niiza-shi, Saitama, 352-8558 Japan
3. Department of Psychology, Kumamoto University, 2-40-1 Kurokami, Chuo-ku, Kumamoto, 860-8555, Japan

*Corresponding authors:

Yoichi Sugita

Department of Psychology, Waseda University,

1-24-1, Toyama, Shinjuku-ku, 162-8644, Japan

E-mail: y.sugita@waseda.jp

Souta Hidaka

Department of Psychology, Rikkyo University,

1-2-26, Kitano, Niiza-shi, Saitama, 352-8558 Japan.

E-mail: hidaka@rikkyo.ac.jp


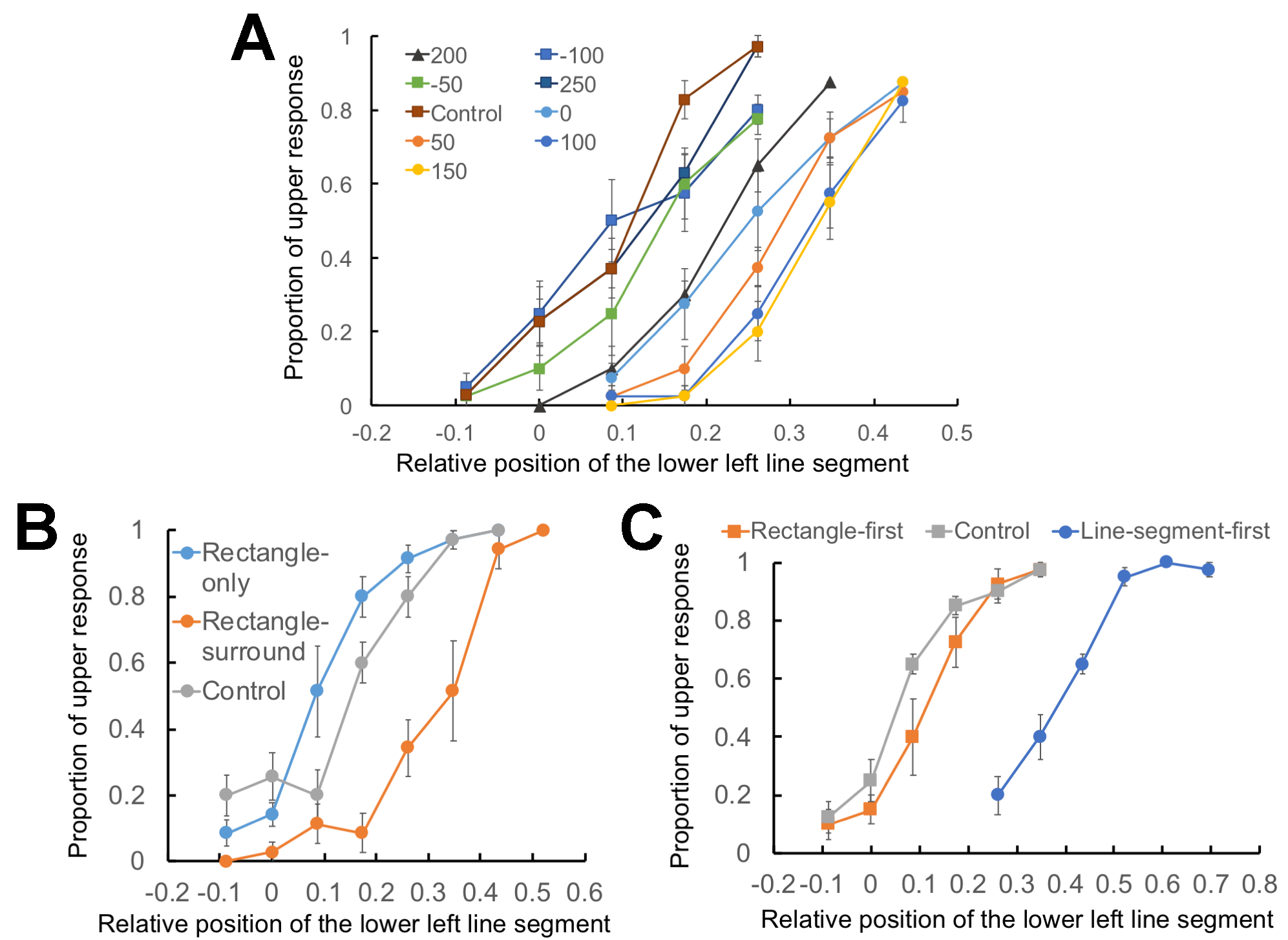


Supplementary figure S1. Psychometric functions of averaged data obtained from (A) Experiment 1, (B) Experiment 2, and (C) Experiment 3. The vertical axis denotes the proportion of upper response for the lower left line segment. The horizontal axis denotes the relative position of the lower left line segment against the objective continuation of the upper right segment. Error bars denote the SEM (N = 7, 7, and 8 in Experiments 1, 2, and 3, respectively).

**Supplementary videos**

Supplementary video S2. A demonstration of the temporal integration of two oblique line segments and a rectangle in the Poggendorff figure. A rectangle is presented followed by two line segments with 100 ms of ISI. Please keep looking at a fixation point. The Poggendorff illusion can be clearly observed.

Supplementary video S3. A demonstration of the temporal integration of the Poggendorff elements with a surrounding moving stimulus. A rectangle moves to left together with the surrounding stimulus after t two line segments are presented. The Poggendorff illusion is perceptible although the line segments do not appear to abut obliquely on the edge of the rectangle.

Supplementary video S4. A demonstration of the temporal integration of the Poggendorff elements along a moving trajectory. The Poggendorff illusion clearly occurs even when two line segments and a rectangle suddenly appear on the moving trajectory.
